# Molecular detection of *Bartonella* spp. in deer ked (*Lipoptena cervi*) in Poland

**DOI:** 10.1186/s13071-017-2413-0

**Published:** 2017-10-16

**Authors:** Tomasz Szewczyk, Joanna Werszko, Żaneta Steiner-Bogdaszewska, Witold Jeżewski, Zdzisław Laskowski, Grzegorz Karbowiak

**Affiliations:** Witold Stefański Institute of Parasitology, Polish Academy of Sciences, Twarda 51/55, 00-818 Warsaw, Poland

**Keywords:** *Bartonella* spp., *Bartonella schoenbuchensis*, *Lipoptena cervi*, *Cervus elaphus*

## Abstract

**Background:**

The bacteria of the genus *Bartonella* are obligate parasites of vertebrates. Their distribution range covers almost the entire world from America, Europe, Asia to Africa and Australia. Some species of *Bartonella* are pathogenic for humans. Their main vectors are blood-sucking arthropods such as fleas, ticks and blood-feeding flies. One such dipteran able to transfer vector-borne pathogens is the deer ked (*Lipoptena cervi*) of the family Hippoboscidae. This species acts as a transmitter of *Bartonella* spp. in cervid hosts in Europe.

**Methods:**

In the present study, 217 specimens of deer ked (*Lipoptena cervi*) were collected from 26 red deer (*Cervus elaphus*) hunted in January 2014. A short fragment (333 bp) of the *rpoB* gene was used as a marker to identify *Bartonella* spp. in deer ked tissue by PCR test. A longer fragment (850 bp) of the *rpoB* gene was amplified from 21 of the positive samples, sequenced and used for phylogenetic analysis.

**Results:**

The overall prevalence of *Lipoptena cervi* infection with *Bartonella* spp. was 75.12% (163/217); 86.67% (104/120) of females and 60.82% (59/97) of males collected from red deer hunted in the Strzałowo Forest District in Poland (53°45′57.03″N, 21°25′17.79″E) were infected. The nucleotide sequences from 14 isolates (*Bartonella* sp. 1) showed close similarity to *Bartonella schoenbuchensis* isolated from moose blood from Sweden (GenBank: KB915628) and human blood from France (GenBank: HG977196); *Bartonella* sp. 2 (5 isolates) and *Bartonella* sp. 3 (one isolate) were similar to *Bartonella* sp. from Japanese sika deer (GenBank: AB703149), and *Bartonella* sp. 4 (one isolate) was almost identical to *Bartonella* sp. isolated from Japanese sika deer from Japan (GenBank: AB703146).

**Conclusions:**

To the best of our knowledge, this is the first report to confirm the presence of *Bartonella* spp. in deer keds (*Lipoptena cervi*) in Poland by molecular methods. *Bartonella* sp. 1 isolates were most closely related to *B. schoenbuchensis* isolated from moose from Sweden and human blood from France. The rest of our isolates (*Bartonella* spp. 2–4) were similar to *Bartonella* spp. isolated from Japanese sika deer from Japan.

**Electronic supplementary material:**

The online version of this article (10.1186/s13071-017-2413-0) contains supplementary material, which is available to authorized users.

## Background

The genus *Bartonella* comprises small, Gram-negative bacteria which act as obligate intracellular parasites of vertebrates. About 30 species, as well as three subspecies, have been described [1]. The zoonotic reservoir for some *Bartonella* species (*B. schoenbuchensis*, *B*. *baciliformis* and *B*. *quintana*), is composed of wild mammals, which usually only possess asymptomatic bacteraemia. The prevalence of bacteremia in wild animals is often very high, ranging from 50 to 95% in selected rodent and ruminant populations [2, 3] and strongly depends on the season [3]. As the reservoir hosts include a wide range of wild mammals, including typically ruminants, rodents and carnivores, the infections are easily spread and have been noted all over the world [2]. A good example is that of ruminant infections: *Bartonella* infection has been recorded in cattle in three different countries (Thailand, Guatemala and Georgia) and in buffalo (*Bubalus bubalis*) from Thailand [4]. The prevalence of *Bartonella* infection in these regions varies between 10 and 90%. Similar investigations in Japan found no infection in cattle, but recorded a prevalence of *Bartonella* sp., 67.5% in Honshu sika deer (*Cervus nippon centralis*) and 50% in Yezo sika deer (*Cervus nippon yesoensis*) [5].

Many *Bartonella* species are considered as human pathogens and causative agents of zoonotic diseases: *B. bacilliformis*, the agent of Carrion’s disease and *Verruga peruna* is the chronic delayed stage of infection; *B*. *quintana*, the agent of trench fever and bacillary angiomatosis; *B. henselae*, the agent of cat scratch disease and of bacillary angiomatosis; *B. clarridgeiae*, *B. elizabethae*, *B*. *vinsonii* subsp. *arupensis*, *B*. *vinsonii* subsp. *berkhoffii*, *B. alsatica*, *B. koehlerae* and *B. washoensis* all cause endocarditis [6–8]. The symptoms of human bartonellosis vary with regard to the bacterial species and general condition of the patient; however, *Bartonella* infection most often manifests as various cardiovascular, neurological and rheumatologic conditions [8, 9].

Bartonellosis has been observed in humans throughout Europe, Asia and North America. One of the most important species is *Bartonella schoenbuchensis*, which is found in cattle, wild animals (such as the cervids) and humans [10–12]. It has been identified in wild living roe deer (*Capreolus capreolus*) in Germany [10], in cattle in France [11] and in humans; *B. schoenbuchensis* was first detected in France [12].

*Bartonella* are transmitted by blood-sucking arthropods, such as lice, flies, fleas and ticks, many of which are emerging pathogens of humans and animals [2, 13–15]. Dehio et al. [16] identified *B. schoenbuchensis* in *Lipoptena cervi* collected from red deer and roe deer. Halos et al. [14] reported the presence of *Bartonella* spp. in hippoboscide flies, and suggested that flies can be a vector for pathogens. Other authors [14, 17] have found *Bartonella* spp*.* in wingless adult deer keds.

*Lipoptena cervi* are blood-sucking parasites which belong to a highly specialized family of louse flies (Diptera: Hippoboscidae) [18, 19]. This species has a Palaearctic distribution and occurs in Europe, Asia and North America; it is known to be present in many countries, including Sweden, Norway, Japan, the USA and Finland [20, 21]. In America, it is regarded as an invasive species, believed to have been transported with deer from Europe in the 1800s. This species was identified in the USA for the first time during the Second World War [18]. In Europe, the most common blood-sucking ectoparasites of mammals belonging to this family are louse fly (*Hippobosca equina*), parasitizing cows and horses, deer keds (*Lipoptena cervi* and *Lipoptena fortisetosa*), parasitizing cervids, and sheep ked (*Melophagus ovinus*), which ectoparasites of sheep [22, 23]. *Lipoptena fortisetosa* in Europe could be introduced with Japanese sika deer (*Cervus nippon*). This cervids were introduced to England in 1860, and their number and range have since increased [24]. From England, the Japanese sika deer were late introduced to Poland, where two species of *Lipoptena* have been identified: *L. cervi* and *L. fortisetosa* [25, 26].

Although the dominant hosts for two species of *Lipoptena* (*L. cervi* and *L*. *fortisetosa*) are cervids (*Alces alces*, *Cervus elaphus*, *Cervus elaphus maral*, *Cervus nippon*, *Dama dama*, *Capreolus capreolus* and *Rangifer tarandus*), the insects can attack a wide range of animals, including bovids (*Ovis aries musimon*, *Bison bonasus*, cattle, *Capra aegagrus hircus* and *Ovis aries*), carnivores (domestic dogs, *Vulpes vulpes* and *Meles meles*) and suids (*Sus scrofa*) [27]. The most important hosts of *Lipoptena cervi* in Europe are red deer (*Cervus elaphus*), roe deer (*Capreolus capreolus*), moose (*Alces alces*) and sika deer (*Cervus nippon*), while Japanese sika deer (*Cervus nippon*) are the predominate host in Japan, and white-tailed deer (*Odocoileus virginianus*), moose (*Alces alces*) and cattle (*Bos taurus*) in North America [21, 28, 29].

Generally, *Lipoptena cervi* is found swarming in large numbers on the host. Deer keds most frequently bite animal hosts; they can land on humans, but rarely bite in this case. Nevertheless, some authors have reported ked bites on humans [30, 31].

The life-cycle of *Lipoptena cervi* begins with free-ranging, winged adult deer keds that search for a suitable host (cervids). The winged deer keds usually suck blood from the host and mate; however, after landing on a host, they can crawl into the fur, shed their wings and become permanently associated with the host. The larvae develop up to stage 3 (L3) in the oviduct and are then deposited in the cervid fur as a white prepupa, which immediately starts to pupate. The fully developed pupa drops to the ground and remains there until August-September, when a new generation of winged adult deer keds can appear [29, 32, 33]. The female can produce to 20–25 larvae per year. *Lipoptena cervi* can overwinter on the host, and most deer keds can live to one year [34].

The winged adult *Lipoptena cervi* are attracted to large moving dark-colored objects when actively searching for a host [35]. *Lipoptena cervi* only fly for short distances and frequently attack accidental hosts such as humans or dogs [32].

*Lipoptena cervi* is a potential vector for other zoonotic pathogens, such as *Anaplasma*, *Borrelia* and *Rickettsia* species [36]. Korhonen et al. [37] showed for the first time transstadial transmission of *Bartonella* spp. in all development stages of deer ked (pupa, unfed adult winged deer ked). These data show vector competence for transmission of *Bartonella* spp. by *Lipoptena cervi*. In America, *Bartonella* sp. has been reported in *Lipoptena cervi* from white-tailed deer in Massachusetts [28], and in *Lipoptena mazamae* from white-tailed deer (*Odocoileus virginianus*) in Georgia and South Carolina (USA) [21]. Additionaly, it has also been suggested that wingless adults of *L. mazamae*, could be transmitted mechanically from female white-tailed deer to their offsprings [38]. *Bartonella* sp. has also been found in ticks (*Ixodes ricinus*) parasitizing roe deer in Poland [39].

The main goal of the study was detection of *Bartonella* spp. DNA in *Lipoptena cervi* by PCR test and verification of prevalence of this infection in adult wingless males and females. Molecular characterization of specimens of *Bartonella* spp. was done by analysis of partial (850 bp) *rpoB* gene sequences.

## Methods

### Sample collection

Living deer keds (*Lipoptena cervi*) were collected from 26 red deer (*Cervus elaphus*) hunted in January 2014 in the Strzałowo Forest District (53°45′57.03″N, 21°25′17.79″E). After collection, all insects were preserved in 70% ethanol for further morphological and molecular processing. Identification of deer ked (*L. cervi*) species and sex were conducted using appropriate identification key [34] and molecular methods to confirm vector species.

### PCR and sequence analyses

In the laboratory, the flies were removed from 70% alcohol and air-dried. The DNA was isolated from whole insect body using the AX Tissue Mini kit (A&A Biotechnology, Gdynia, Poland) according to the manufacturer’s protocol.

For PCR test the primers rpoR and rpoF were used. These primers amplify a 333 bp fragment of the *rpoB* gene of *Bartonella* spp. PCR reactions were conducted according to Paziewska et al. [40]. 21 of 163 positive samples were used to obtain a longer (850 bp) fragment of the *rpoB* gene with second set of primers (1400F and 2300R) [41] (Table 1).

**Table 1:** List of primers sequences used in this study

Primer	Sequence (5′-3′)	Reference
L700F	AAAGTTTAACCTGCCCACTGAT	This study
L1213R	CTGAACTCAGATCACGTAAGAAT	This study
rpoR	CGCATTATGGTCGTATTTGTCC	Paziewska et al. [40]
rpoF	GCACGATTYGCATCATCATTTTCC	Paziewska et al. [40]
1400F	CGCATTGGCTTACTTCGTATG	Renesto et al. [41]
2300R	GTAGACTGATTAGAACGCTG	Renesto et al. [41]

PCR reactions were conducted in a 50 μl reaction mixture containing 2 μl of DNA template, 0.5 U (0.1 μl) of RUN *Taq* polymerase (A&A Biotechnology, Gdynia, Poland), 1 μl of dNTPs (10 mM), 0.5 μl of each primer (20 mM), and 5 μl of 10× *Taq* DNA polymerase buffer (pH 8.6, 25 mM MgCl_2_). In the negative control, nuclease-free water was added to the PCR mix instead of the tested DNA.

DNA amplification (1400F/2300R) was performed using the DNA Engine PTC-200 Thermal Cycler (BioRad, Hercules, USA) using the following program: initial denaturation was performed at 94 °C for 5 min, followed by 35 cycles of denaturation at 95 °C for 10 s, annealing at 60 °C for 10 s and extension at 72 °C for 60 s. The final extension was performed at 72 °C for 7 min and then kept at 10 °C in a thermocycler*.*

The PCR products were visualized on a 1.0% agarose gel stained with ethidium bromide. Visualization was performed using ChemiDoc, MP Lab software (Imagine, BioRad, Hercules, USA). The resulting product was compared using the Nova 100 bp DNA Ladder Novazym (Poznań, Poland). The PCR amplicons were purified using a QIAEX II Gel Extraction Kit (Qiagen, Hilden, Germany), sequenced in both directions by Genomed (Poland) and contiguous sequences assembled using ContigExpress, Vector NTI Advance 11.0 (Invitrogen Life Technologies, New York, USA). The derived sequences were submitted to the GenBank database under the accession numbers MF580655–MF580675.

To confirm the morphological species determination of *Lipoptena cervi*, DNA from four specimens (positive in *Bartonella* spp. test and used in *Bartonella* isolate analysis), L700F and L1213R primers were used to amplify and sequence 412 bp fragments of 16S rDNA (Table 1).

In order to perform PCR amplification, the following mixture reaction was used: 4 μl of DNA extract was added to 46 μl of reaction mixture consisting of 0.1 μl of Allegro Taq Polymerase DNA (5 U/μl) (Poznań, Poland), 0.5 μl dNTPs (10 mM), 1 μl of each primer (20 mM), 5 μl of 10× *Taq* DNA polymerase buffer (pH 8.6, 25 mM MgCl_2_), and 38.4 μl of deionized water.

DNA amplification (L700F/L1213R) was performed using the DNA Engine PTC-200 Thermal Cycler (BioRad, Hercules, USA) using the following program:an initial denaturation was performed at 92 °C for 3 min, followed by 35 cycles of denaturation at 95 °C for 10 s, annealing at 60 °C for 10 s and extension at 72 °C for 30 s. The final extension was performed at 72 °C for 5 min and then kept at 12 °C in a thermocycler*.*

### Phylogenetic analyses

We used Bayesian inference (BI) analysis with MrBayes version 3.2 [42]. Analysis of partial *rpoB* gene sequence data was based on an alignment of 804 bp (268 amino acids) using the GTR + I + G model. The GTR models were chosen on the basis of jModelTest version 2.1.4 [43, 44] using the Akaike information criterion.

## Results

In total, 217 deer keds were collected from 26 red deer (*Cervus elaphus*). All insects were identified as *Lipoptena cervi* using morphological features [34]. The prevalence of *Bartonella* spp. infection was 75.12% (163/217) by PCR test. In the tested group of *L. cervi*, a greater proportion of females (86.67%) was found to be positive for *Bartonella* spp. than males (60.82%).

### PCR, sequence and molecular analyses

Fourteen sequences (*Bartonella* sp. 1: MF580662–MF580675) obtained in this study share over 99% similarity with *B. schoenbuchensis* isolated from moose blood from Sweden (GenBank: KB915628) and from human blood from France (GenBank: HG977196). Five sequences *Bartonella* sp. 2 (MF580657–MF580661) showed 94.6% similarity with *Bartonella* sp. 3, and 94.4% with *Bartonella* sp. from Japanese sika deer from Japan (AB703149). *Bartonella* sp. 3 (MF580656) showed 99.7% similarity with another samples isolated from Japanese sika deer from Wakayama Prefecture Japan (AB703149). *Bartonella* sp. 4 (MF580655) was 99,7% similar with *Bartonella* sp. isolated from Japanese sika deer (*Cervus nippon* *centralis*) from Nara Prefecture Japan (AB703146). These results are summarized in Table 2 and Additional file 1: Table S1.

**Table 2:** *Bartonella* spp. used in the phylogenetic analysis

Isolate/sequence ID	Species	Host	Source	Country of isolation
MF580662–MF580675	*Bartonella* sp. 1	*Lipoptena cervi*	whole body	Poland
MF580657–MF580661	*Bartonella* sp. 2	*Lipoptena cervi*	whole body	Poland
MF580656	*Bartonella* sp. 3	*Lipoptena cervi*	whole body	Poland
MF580655	*Bartonella* sp. 4	*Lipoptena cervi*	whole body	Poland
DQ356077	*Bartonella bovis*	bovine	blood	Italy
EF432062	*B. bovis*	cow	valve (heart)	France
KR733195	*B. bovis*	cattle	blood	Malaysia
KR733194	*B. bovis*	cattle	blood	Malaysia
KF218224	*B. bovis*	water buffalo	blood	Thailand
KF218220	*B. bovis*	cattle	blood	Thailand
KF218218	*B. bovis*	cattle	blood	Guatemala
KF218217	*B. bovis*	cattle	blood	France
HM167505	*Bartonella capreoli*	moose	blood	USA
AB703143	*B. capreoli*	Japanese sika deer	blood	Japan
AB703142	*B. capreoli*	Japanese sika deer	blood	Japan
AB703149	*Bartonella* sp.	Japanese sika deer	blood	Japan
AB703146	*Bartonella* sp.	Japanese sika deer	blood	Japan
AB703145	*Bartonella* sp.	Japanese sika deer	blood	Japan
KB915628	*Bartonella schoenbuchensis*	moose	blood	Sweden
KM215709	*Bartonella chomelii*	cattle	blood	Spain
KM215710	*Bartonella chomelii*	cattle	blood	Spain
JN646664	*Bartonella chomelii*	cattle	blood	New Caledonia
KJ909808	*Bartonella bovis*	cattle	blood	Israel
AB703148	*Bartonella* sp.	Japanese sika deer	blood	Japan
AB703144	*Bartonella* sp.	Japanese sika deer	blood	Japan
HG977196	*Bartonella schoenbuchensis*	human	blood	France
CP019789	*Bartonella schoenbuchensis*	European roe deer	blood	Germany

Four 16S rDNA sequences of deer ked were obtained, which were identical with *Lipoptena cervi* collected in the Czech Republic (AF322437). The sequences derived during this study were submitted to the GenBank database under the accession numbers MF541726–MF541729.

## Discussion

Our findings indicate the presence of *Bartonella* species in deer keds (*Lipoptena cervi*) obtained from red deer (*Cervus elaphus*). Dehio et al. [16] noted that *L. cervi* appears to be a natural reservoir supporting the transmission of *Bartonella schoenbuchensis*. The largest group of isolates (*Bartonella* sp. 1) showed closed similarity with *Bartonella schoenbuchensis*. Our data indicate a high prevalence of *Bartonella* spp. A prevalence of 75.12% was recorded in the whole tested group, a result similar to global data; however, higher values were observed in Finland (90%) and France (94%) [14, 37].

To the best of our knowledge, the present paper is the first report in Poland to identify differences in the prevalence of *Bartonella* spp. infection in male and female deer keds. Females were more frequently infected than males; 86.67% of wingless females were infected by *Bartonella*, similar to a study from Hungary (76.0%), while only 60.82% of wingless males were infected, also similar to a result in Hungary (58%) [45].

The reason for such a difference in infection prevalence is not clear; however, it might be associated with the multiple bites needed for larvae production (20–25 larvae per year) [34]. De Bruin et al. [45] suggested that *Bartonella* species are able to colonize or survive more efficiently as females than males, but the molecular mechanism for this remains unknown. The same authors [45] suggested that *Bartonella* spp. infection in female deer keds might lead to more female offspring than uninfected females, possibly resulting in the observed asymmetry in the female:male ratio of infected individuals.

By transmitting pathogens, *Lipoptena cervi* can be potentially dangerous for animals and humans. A study conducted in Finland found that *L. cervi* can transmit some pathogens by biting, and this is associated with hair-loss epizootics in moose [17]. Since the 1970s, in Finland an increasing number of cases of recurrent, and occasionally long-lasting, dermatitis associated with deer keds bites has been observed [46].

Fourteen of the sequences (*Bartonella* sp. 1, see Table 2.) isolated from deer keds demonstrated 99.2% homology with a *Bartonella schoenbuchensis* sample isolated from moose in Sweden (KB915628). *Bartonella schoenbuchensis* could be found in vectors, such as *L. cervi* or *L. mazamae*, or in definitive hosts like cattle, wild ruminants and humans [10, 11, 16]. It is difficult to see how the relationship between *B. schoenbuchensis* and deer ectoparasites may relate to similar cases in humans. *Lipoptena cervi* has fed on humans under experimental conditions and in the natural environment [18, 47]. As humans may be infected with *B. schoenbuchensis* during occasional biting by *L. cervi*, hunters, forestry workers and cross-country runners, among others, are at increased risk of infection [16]. Although *L. cervi* has not yet been definitively demonstrated to transmit *B. schoenbuchensis* through bites, it is possible that hunters are at risk to infection of *Bartonella schoenbuchensis* or *Anaplasma phagocytophilum* following exposure to deer blood [48].

In Massachusetts, a *Bartonella* sp. similar to *B. schoenbuchensis* has been found in *Lipoptena mazamae* [21], which suggests that *Lipoptena* species can extend the range of *B. schoenbuchensis*. Our phylogenetic analysis of the partial *rpoB* gene sequences found that our samples were in the same clade as other *B. schoenbuchensis* isolates from moose (KB915628) and human (HG977196). *Bartonella schoenbuchensis* (strain closely related to *B. schoenbuchensis* strain R1–86.6%, *gltA* gene) was previously reported from roe deer (*C. capreolus*) in Poland [49].

**Fig. 1:**
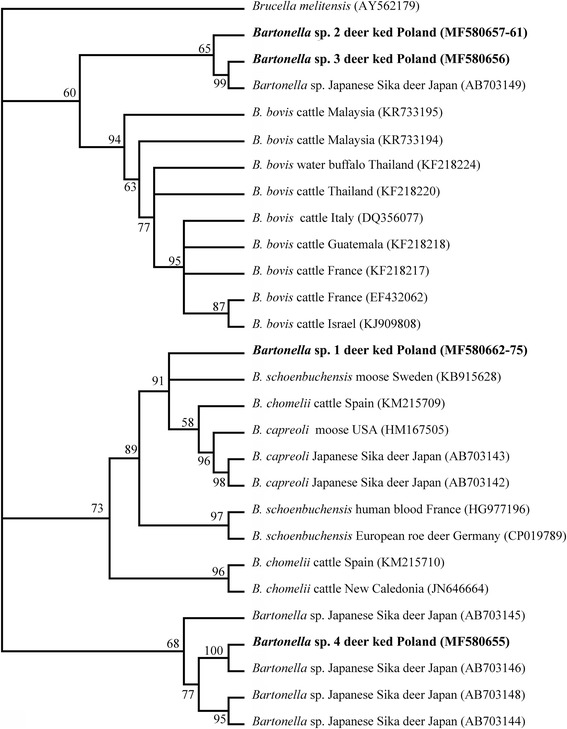
Phylogenetic tree of *Bartonella* spp., constructed by Bayesian inference (BI) analysis using MrBayes version 3.2. For BI codon analysis (nucmodel = codon), the GTR + I + G model was chosen based on jModelTest version 2.1.4 [43, 44] using Akaike Information Criterion. Analysis was run for 8,000,000 generations, with 2,000,000 generations discarded as ‘burn-in’. Hosts, country and GenBank accession numbers of origin are shown. Nodal support is indicated as Bayesian posterior probabilities. Sequence from *Brucella melitensis* (AY562179) was used as outgroup. Sequences generated in this study are shown in bold

*Bartonella* sp. 2 (5 isolates) and *Bartonella* sp. 3 (MF580656) demonstrated high similarity to sequences obtained from Japanese sika deer from Japan (AB703149, 94.6% and 99.7% similarity, respectively. These *Bartonella* isolates are related with the *B. bovis* clade in our phylogenetic analysis (Fig. 1).

*Bartonella* sp. 4 (MF580655) is very similar (99.7%) to *Bartonella* sp. isolates collected from Japanese sika deer (AB703146, see Fig. 1) and in our phylogenetic analyses these isolates are associated with other *Bartonella* sp. isolates from Japanese sika deer and formed a distinct clade with *Bartonella* spp. isolated from Japanese sika deer. Probably “japanese” *Bartonella* isolates were introducted to Poland. This observation suggests that the strain of *Bartonella* sp. bacteria identified in the present study is derived from Japanese sika deer introduced to Europe by vectors to environment, indicating that this Asian strains could be spread by *L. cervi* to European red deer (*Cervus elaphus*).

Some Japanese *Bartonella* sp. samples, isolated from different prefectures, comprise a group distinct from other samples of *Bartonella* on GenBank; however, further testing is required utilizing other genes, including *gltA* and *ftsZ*, to confirm whether they could be regarded as a distinct species.

## Conclusions

Our data confirm that *Bartonella* spp. can be transmitted by deer ked in central Europe, and the prevalence of this pathogen is very high. In this area, *Lipoptena cervi* are infected by various species of the genus *Bartonella*.

## Additional files

Additional file 1: Table S1. Pairwise comparison of partial (804 bp) mitochondrial gene *rpoB* DNA and amino acid sequences variability among species/sequences of *Bartonella* used in phylogenetical analysis. Above diagonal: number of variable sites in the 268 amino acid gene *rpoB* sequences. Below diagonal: number of variable sites in the nucleotides gene *rpoB* sequences. The percentage of variable sites for each gene fragment between 2 species/sequences is given in parenthesis. Each species isolates/sequence information are provided in Table 2. (DOCX 23 kb)
