# Supplementary material for: Molecular detection of Bartonella spp. in deer ked (Lipoptena cervi) in Poland
Source: Parasit Vectors. 2017 Oct 16;10:487. doi: 10.1186/s13071-017-2413-0 (PMC5644074; doi:10.1186/s13071-017-2413-0)
Supplement: Supplementary file 1 — Pairwise comparison of partial (804 bp) mitochondrial gene rpoB DNA and amino acid sequences variability among species/sequences of Bartonella used in phylogenetical analysis. Above diagonal: number of variable sites in the 268 amino acid gene rpoB sequences. Below diagonal: number of variable sites in the nucleotides gene rpoB sequences. The percentage of variable sites for each gene fragment between 2 species/sequences is given in parenthesis. Each species isolates/sequence information are provided in Table 2. (DOCX 23 kb) [file 13071_2017_2413_MOESM1_ESM.docx]

| Species/sequences | 1 | 2 | 3 | 4 | 5 | 6 | 7 | 8 | 9 | 10 | 11 | 12 | 13 | 14 | 15 | 16 | 17 | 18 | 19 | 20 | 21 | 22 | 23 | 24 | 25 | 26 | 27 |
| --- | --- | --- | --- | --- | --- | --- | --- | --- | --- | --- | --- | --- | --- | --- | --- | --- | --- | --- | --- | --- | --- | --- | --- | --- | --- | --- | --- |
| 1. *B.* sp. 1 (MF580662-75) | **ID** | 2 (0.8) | 1 (0.4) | 2 (0.8) | 0 (0.00) | 0 (0.00) | 0 (0.00) | 2 (0.8) | 0 (0.00) | 0 (0.00) | 0 (0.00) | 0 (0.00) | 0 (0.00) | 0 (0.00) | 1 (0.4) | 2 (0.8) | 1 (0.4) | 1 (0.4) | 2 (0.8) | 1 (0.4) | 0 (0.00) | 1 (0.4) | 2 (0.8) | 1 (0.4) | 0 (0.00) | 1 (0.4) | 0 (0.00) |
| 2. *B.* sp. 2 (MF580657-61) | 42 (5.3) | **ID** | 1 (0.4) | 2 (0.8) | 2 (0.8) | 2 (0.8) | 2 (0.8) | 2 (0.8) | 2 (0.8) | 2 (0.8) | 2 (0.8) | 2 (0.8) | 2 (0.8) | 2 (0.8) | 3 (1.2) | 2 (0.8) | 1 (0.4) | 1 (0.4) | 2 (0.8) | 3 (1.2) | 2 (0.8) | 1 (0.4) | 2 (0.8) | 1 (0.4) | 2 (0.8) | 3 (1.2) | 2 (0.8) |
| 3. *B.* sp. 3 (MF580656) | 37 (4.7) | 43 (5.4) | **ID** | 1 (0.4) | 1 (0.4) | 1 (0.4) | 1 (0.4) | 1 (0.4) | 1 (0.4) | 1 (0.4) | 1 (0.4) | 1 (0.4) | 1 (0.4) | 1 (0.4) | 2 (0.8) | 1 (0.4) | 0 (0.00) | 0 (0.00) | 1 (0.4) | 2 (0.8) | 1 (0.4) | 0 (0.00) | 1 (0.4) | 0 (0.00) | 1 (0.4) | 2 (0.8) | 1 (0.4) |
| 4. *B.* sp. 4 (MF580655) | 29 (3.7) | 53 (6.6) | 42 (5.3) | **ID** | 2 (0.8) | 2 (0.8) | 2 (0.8) | 2 (0.8) | 2 (0.8) | 2 (0.8) | 2 (0.8) | 2 (0.8) | 2 (0.8) | 2 (0.8) | 3 (1.2) | 2 (0.8) | 1 (0.4) | 1 (0.4) | 0 (0.00) | 3 (1.2) | 2 (0.8) | 1 (0.4) | 2 (0.8) | 1 (0.4) | 2 (0.8) | 3 (1.2) | 2 (0.8) |
| 5. *B. bovis* (DQ356077) | 45 (5.6) | 52 (6.5) | 52 (6.5) | 52 (6.5) | **ID** | 0 (0.00) | 0 (0.00) | 2 (0.8) | 0 (0.00) | 0 (0.00) | 0 (0.00) | 0 (0.00) | 0 (0.00) | 0 (0.00) | 1 (0.4) | 2 (0.8) | 1 (0.4) | 1 (0.4) | 2 (0.8) | 1 (0.4) | 0 (0.00) | 1 (0.4) | 2 (0.8) | 1 (0.4) | 0 (0.00) | 1 (0.4) | 0 (0.00) |
| 6*. B. bovis* (EF432062) | 43 (5.4) | 50 (6.3) | 50 (6.3) | 50 (6.3) | 2 (0.3) | **ID** | 0 (0.00) | 2 (0.8) | 0 (0.00) | 0 (0.00) | 0 (0.00) | 0 (0.00) | 0 (0.00) | 0 (0.00) | 1 (0.4) | 2 (0.8) | 1 (0.4) | 1 (0.4) | 2 (0.8) | 1 (0.4) | 0 (0.00) | 1 (0.4) | 2 (0.8) | 1 (0.4) | 0 (0.00) | 1 (0.4) | 0 (0.00) |
| 7. *B. bovis* (KJ909808) | 43 (5.4) | 50 (6.3) | 50 (6.3) | 51 (6.4) | 3 (0.4) | 1 (0.2) | **ID** | 2 (0.8) | 0 (0.00) | 0 (0.00) | 0 (0.00) | 0 (0.00) | 0 (0.00) | 0 (0.00) | 1 (0.4) | 2 (0.8) | 1 (0.4) | 1 (0.4) | 2 (0.8) | 1 (0.4) | 0 (0.00) | 1 (0.4) | 2 (0.8) | 1 (0.4) | 0 (0.00) | 1 (0.4) | 0 (0.00) |
| 8*. B. chomelii* (KM215710) | 16 (2.0) | 45 (5.6) | 36 (4.5) | 30 (3.8) | 48 (6.0) | 46 (5.8) | 46 (5.8) | **ID** | 2 (0.8) | 2 (0.8) | 2 (0.8) | 2 (0.8) | 2 (0.8) | 2 (0.8) | 3 (1.2) | 2 (0.8) | 1 (0.4) | 1 (0.4) | 2 (0.8) | 3 (1.2) | 2 (0.8) | 1 (0.4) | 2 (0.8) | 1 (0.4) | 2 (0.8) | 3 (1.2) | 2 (0.8) |
| 9. *B bovis* (KR733195) | 40 (5.0) | 49 (6.1) | 51 (6.4) | 47 (5.9) | 5 (0.7) | 5 (0.7) | 6 (0.8) | 43 (5.4) | **ID** | 0 (0.00) | 0 (0.00) | 0 (0.00) | 0 (0.00) | 0 (0.00) | 1 (0.4) | 2 (0.8) | 1 (0.4) | 1 (0.4) | 2 (0.8) | 1 (0.4) | 0 (0.00) | 1 (0.4) | 2 (0.8) | 1 (0.4) | 0 (0.00) | 1 (0.4) | 0 (0.00) |
| 10. *B. bovis* (KR733194) | 41 (5.1) | 50 (6.3) | 50 (6.3) | 48 (6.0) | 4 (0.5) | 4 (0.5) | 5 (0.7) | 44 (5.5) | 1 (0.2) | **ID** | 0 (0.00) | 0 (0.00) | 0 (0.00) | 0 (0.00) | 1 (0.4) | 2 (0.8) | 1 (0.4) | 1 (0.4) | 2 (0.8) | 1 (0.4) | 0 (0.00) | 1 (0.4) | 2 (0.8) | 1 (0.4) | 0 (0.00) | 1 (0.4) | 0 (0.00) |
| 11. *B. bovis* (KF218224) | 42 (5.3) | 51 (6.4) | 51 (6.4) | 47 (5.9) | 5 (0.7) | 5 (0.7) | 6 (0.8) | 44 (5.5) | 4 (0.5) | 3 (0.4) | **ID** | 0 (0.00) | 0 (0.00) | 0 (0.00) | 1 (0.4) | 2 (0.8) | 1 (0.4) | 1 (0.4) | 2 (0.8) | 1 (0.4) | 0 (0.00) | 1 (0.4) | 2 (0.8) | 1 (0.4) | 0 (0.00) | 1 (0.4) | 0 (0.00) |
| 12*. B. bovis* (KF218220) | 43 (5.4) | 52 (6.5) | 52 (6.5) | 49 (6.1) | 5 (0.7) | 5 (0.7) | 6 (0.8) | 46 (5.8) | 4 (0.5) | 3 (0.4) | 4 (0.5) | **ID** | 0 (0.00) | 0 (0.00) | 1 (0.4) | 2 (0.8) | 1 (0.4) | 1 (0.4) | 2 (0.8) | 1 (0.4) | 0 (0.00) | 1 (0.4) | 2 (0.8) | 1 (0.4) | 0 (0.00) | 1 (0.4) | 0 (0.00) |
| 13*. B. bovis* (KF218218) | 44 (5.5) | 51 (6.4) | 51 (6.4) | 51 (6.4) | 1 (0.2) | 1 (0.2) | 2 (0.3) | 47 (5.9) | 4 (0.5) | 3 (0.4) | 4 (0.5) | 4 (0.5) | **ID** | 0 (0.00) | 1 (0.4) | 2 (0.8) | 1 (0.4) | 1 (0.4) | 2 (0.8) | 1 (0.4) | 0 (0.00) | 1 (0.4) | 2 (0.8) | 1 (0.4) | 0 (0.00) | 1 (0.4) | 0 (0.00) |
| 14. *B. bovis* (KF218217) | 45 (5.6) | 53 (6.6) | 53 (6.6) | 53 (6.6) | 3 (0.4) | 3 (0.4) | 4 (0.5) | 48 (6.0) | 6 (0.8) | 5 (0.7) | 6 (0.8) | 6 (0.8) | 2 (0.3) | **ID** | 1 (0.4) | 2 (0.8) | 1 (0.4) | 1 (0.4) | 2 (0.8) | 1 (0.4) | 0 (0.00) | 1 (0.4) | 2 (0.8) | 1 (0.4) | 0 (0.00) | 1 (0.4) | 0 (0.00) |
| 15*. B. capreoli* (HM167505) | 12 (1.5) | 44 (5.5) | 41 (5.1) | 31 (3.9) | 46 (5.8) | 44 (5.5) | 44 (5.5) | 20 (2.5) | 41 (5.1) | 42 (5.3) | 43 (5.4) | 44 (5.5) | 45 (5.6) | 46 (5.8) | **ID** | 3 (1.2) | 2 (0.8) | 2 (0.8) | 3 (1.2) | 2 (0.8) | 1 (0.4) | 2 (0.8) | 3 (1.2) | 2 (0.8) | 1 (0.4) | 2 (0.8) | 1 (0.4) |
| 16. *B. capreoli* (AB703143) | 14 (1.8) | 44 (5.5) | 41 (5.1) | 33 (4.2) | 46 (5.8) | 44 (5.5) | 44 (5.5) | 18 (2.3) | 41 (5.1) | 42 (5.3) | 43 (5.4) | 44 (5.5) | 45 (5.6) | 46 (5.8) | 12 (1.5) | **ID** | 1 (0.4) | 1 (0.4) | 2 (0.8) | 2 (0.8) | 2 (0.8) | 1 (0.4) | 2 (0.8) | 1 (0.4) | 2 (0.8) | 3 (1.2) | 2 (0.8) |
| 17*. B. capreoli* (AB703142) | 13 (1.7) | 43 (5.4) | 42 (5.3) | 32 (4.0) | 47 (5.9) | 45 (5.6) | 45 (5.6) | 19 (2.4) | 42 (5.3) | 43 (5.4) | 44 (5.5) | 45 (5.6) | 46 (5.8) | 47 (5.9) | 9 (1.2) | 3 (0.4) | **ID** | 1 (0.4) | 1 (0.4) | 2 (0.8) | 1 (0.4) | 0 (0.00) | 1 (0.4) | 0 (0.00) | 1 (0.4) | 2 (0.8) | 1 (0.4) |
| 18. *B.* sp. (AB703149) | 37 (4.7) | 45 (5.6) | 2 (0.3) | 42 (5.3) | 52 (6.5) | 50 (6.3) | 50 (6.3) | 37 (4.7) | 51 (6.4) | 50 (6.3) | 51 (6.4) | 52 (6.5) | 51 (6.4) | 53 (6.6) | 41 (5.1) | 41 (5.1) | 42 (5.3) | **ID** | 1 (0.4) | 2 (0.8) | 1 (0.4) | 0 (0.00) | 1 (0.4) | 0 (0.00) | 1 (0.4) | 2 (0.8) | 1 (0.4) |
| 19. *B.* sp. (AB703146) | 29 (3.7) | 55 (6.9) | 44 (5.5) | 2 (0.3) | 54 (6.8) | 52 (6.5) | 53 (6.6) | 30 (3.8) | 49 (6.1) | 50 (6.3) | 49 (6.1) | 51 (6.4) | 53 (6.6) | 55 (6.9) | 31 (3.9) | 33 (4.2) | 32 (4.0) | 44 (5.5) | **ID** | 3 (1.2) | 2 (0.8) | 1 (0.4) | 2 (0.8) | 1 (0.4) | 2 (0.8) | 3 (1.2) | 2 (0.8) |
| 20. *B.* sp. (AB703145) | 21 (2.7) | 47 (5.9) | 40 (5.0) | 22 (2.8) | 47 (5.9) | 45 (5.6) | 45 (5.6) | 22 (2.8) | 42 (5.3) | 43 (5.4) | 44 (5.5) | 46 (5.8) | 46 (5.8) | 48 (6.0) | 23 (2.9) | 25 (3.2) | 26 (3.3) | 40 (5.0) | 22 (2.8) | **ID** | 1 (0.4) | 2 (0.8) | 3 (1.2) | 2 (0.8) | 1 (0.4) | 2 (0.8) | 1 (0.4) |
| 21. *B. chomelii* (KM215709) | 5 (0.7) | 45 (5.6) | 40 (5.0) | 28 (3.5) | 47 (5.9) | 45 (5.6) | 45 (5.6) | 17 (2.2) | 42 (5.3) | 43 (5.4) | 44 (5.5) | 45 (5.6) | 46 (5.8) | 47 (5.9) | 11 (1.4) | 13 (1.7) | 12 (1.5) | 40 (5.0) | 28 (3.5) | 18 (2.3) | **ID** | 1 (0.4) | 2 (0.8) | 1 (0.4) | 0 (0.00) | 1 (0.4) | 0 (0.00) |
| 22. *B. chomelii* (JN646664) | 15 (1.9) | 46 (5.8) | 37 (4.7) | 29 (3.7) | 45 (5.6) | 43 (5.4) | 43 (5.4) | 3 (0.4) | 40 (5.0) | 41 (5.1) | 41 (5.1) | 43 (5.4) | 44 (5.5) | 45 (5.6) | 21 (2.7) | 17 (2.2) | 18 (2.3) | 38 (4.8) | 29 (3.7) | 21 (2.7) | 16 (2.0) | **ID** | 1 (0.4) | 0 (0.00) | 1 (0.4) | 2 (0.8) | 1 (0.4) |
| 23*. B*. sp. (AB703148) | 26 (3.3) | 48 (6.0) | 43 (5.4) | 17 (2.2) | 46 (5.8) | 44 (5.5) | 45 (5.6) | 25 (3.2) | 41 (5.1) | 42 (5.3) | 43 (5.4) | 45 (5.6) | 45 (5.6) | 47 (5.9) | 28 (3.5) | 28 (3.5) | 29 (3.7) | 45 (5.6) | 17 (2.2) | 15 (1.9) | 23 (2.9) | 24 (3.0) | **ID** | 1 (0.4) | 2 (0.8) | 3 (1.2) | 2 (0.8) |
| 24. *B*. sp. (AB703144) | 26 (3.3) | 48 (6.0) | 43 (5.4) | 17 (2.2) | 46 (5.8) | 44 (5.5) | 45 (5.6) | 25 (3.2) | 41 (5.1) | 42 (5.3) | 43 (5.4) | 45 (5.6) | 45 (5.6) | 47 (5.9) | 28 (3.5) | 28 (3.5) | 29 (3.7) | 45 (5.6) | 17 (2.2) | 15 (1.9) | 23 (2.9) | 24 (3.0) | 2 (0.3) | **ID** | 1 (0.4) | 2 (0.8) | 1 (0.4) |
| 25*. B. schoenbuchensis* (HG977196) | 5 (0.7) | 43 (5.4) | 40 (5.0) | 28 (3.5) | 45 (5.6) | 43 (5.4) | 43 (5.4) | 15 (1.9) | 40 (5.0) | 41 (5.1) | 42 (5.3) | 43 (5.4) | 44 (5.5) | 45 (5.6) | 15 (1.9) | 15 (1.9) | 14 (1.8) | 40 (5.0) | 28 (3.5) | 18 (2.3) | 8 (1.0) | 12 (1.5) | 23 (2.9) | 23 (2.9) | **ID** | 1 (0.4) | 0 (0.00) |
| 26*. B. schoenbuchensis* (KB915628) | 6 (0.8) | 44 (5.5) | 39 (4.9) | 33 (4.2) | 48 (6.0) | 46 (5.8) | 46 (5.8) | 16 (2.0) | 43 (5.4) | 44 (5.5) | 45 (5.6) | 46 (5.8) | 47 (5.9) | 48 (6.0) | 14 (1.8) | 14 (1.8) | 13 (1.7) | 39 (4.9) | 33 (4.2) | 23 (2.9) | 7 (0.9) | 15 (1.9) | 28 (3.5) | 28 (3.5) | 9 (1.2) | **ID** | 1 (0.4) |
| 27. *B. schoenbuchensis* (CP019789) | 5 (0.7) | 43 (5.4) | 40 (5.0) | 28 (3.5) | 45 (5.6) | 43 (5.4) | 43 (5.4) | 15 (1.9) | 40 (5.0) | 41 (5.1) | 42 (5.3) | 43 (5.4) | 44 (5.5) | 45 (5.6) | 15 (1.9) | 15 (1.9) | 14 (1.8) | 40 (5.0) | 28 (3.5) | 18 (2.3) | 8 (1.0) | 12 (1.5) | 23 (2.9) | 23 (2.9) | 0 (0.0) | 9 (1.2) | **ID** |

Pairwise comparison of partial (804 bp) mitochondrial gene *rpoB* DNA and amino acid sequences variability among species/sequences of *Bartonella* used in phylogenetical analysis. Above diagonal = number of variable sites in the 268 amino acid gene *rpoB* sequences. Below diagonal = number of variable sites in the nucleotides gene *rpoB* sequences. Percentage of variable sites for each gene fragment between 2 species/sequences is given in parenthesis. Each species isolates/sequences information are posted in table 2.
